# Immunosuppression in Experimental Chagas Disease Is Mediated by an Alteration of Bone Marrow Stromal Cell Function During the Acute Phase of Infection

**DOI:** 10.3389/fimmu.2018.02794

**Published:** 2018-12-10

**Authors:** Uwe Müller, Günter A. Schaub, Horst Mossmann, Gabriele Köhler, Rita Carsetti, Christoph Hölscher

**Affiliations:** ^1^Max Planck Institute for Immunobiology and Epigenetics, Freiburg, Germany; ^2^Institute of Immunology, Veterinary Medicine, University Leipzig Leipzig, Germany; ^3^Department of Animal Ecology, Evolution, and Biodiversity, Ruhr-Universität-Bochum, Bochum, Germany; ^4^Department of Pathology, University of Freiburg, Freiburg, Germany; ^5^Infection Immunology, Research Center Borstel, Borstel, Germany

**Keywords:** *T. cruzi*, B cell depletion, bone marrow, stromal cells, immunosuppression

## Abstract

After infection with *Trypanosoma cruzi*, the etiologic agent of Chagas disease, immunosuppression, and apoptosis of mature lymphocytes contribute to the establishment of the parasite in the host and thereby to persistence and pathology in the chronic stage of infection. In a systemic mouse model of experimental Chagas disease, we have demonstrated a strong depletion of mature B cells in the spleen during the first 2 weeks of infection. Remarkably, the decrease in this cell population commenced already in the bone marrow from infected mice and was a concomitant of an increased apoptosis in pro- and pre-B cell populations. Pro- and pre-B cells in the bone marrow showed a significant reduction accompanied by a functional disturbance of bone marrow-derived stromal cells resulting in diminished levels of IL-7, an essential factor for the development of B cell precursors. *Ex vivo*, stromal cells isolated from the bone marrow of infected mice had a strikingly impaired capacity to maintain the development of pro- and pre-B cells obtained from uninfected animals. Together, the reduction of an active humoral immune response during acute Chagas disease suggests to be an initial immune evasion mechanism of the parasite to establish persistent infection. Therefore, prevention of B cell depletion by rescuing the stromal cells during this early phase, could give rise to new therapeutic approaches.

## Introduction

Antigen-specific B cells are in addition to T cell-mediated immune responses (1–4) essential for controlling infection with the protozoan parasite *Trypanosoma cruzi*, the etiologic agent of Chagas disease (5, 6). Therefore, *T. cruzi* evolved mechanisms to escape a protective B cell response by inducing a strong polyclonal B cell activation (7, 8), B cell anergy (9), and apoptosis (10). In this respect, bone marrow hypoplasia has been described as another cause for B cell depletion after infection with *T. cruzi* (11).

In the bone marrow, hematopoietic stem cells are essential for lymphocyte development, in case of B cells they are generated from multipotent progenitor cells, that rises from these hematopoietic stem cells, but have limited potential and lost the stem-cell properties (12). The differentiation pathway from multipotent progenitor cells to mature B cells can be divided into several stages (13). Progenitor (pro-) B cells start to rearrange the Ig H chain locus and differentiate via precursor (pre-) B I cells into pre-B II cells carrying the μH chain in the cytoplasm which can be assembled into a functional pre-B cell receptor (BCR) (14). Presumably, successful rearrangement of the H chain and a correctly assembled pre-BCR allow pre-B II cells to proliferate (15). After rearrangement of the L chain locus, pre-B II cells become immature B cells leave the bone marrow at the transitional B cell stage and complete their final development into mature B cells in the periphery (16).

Bone marrow stromal cells are essential components of the hematopoietic microenvironment and are absolutely required for the maintenance of hemotopoietic stem cells (17) and the development of B cells (18). Stromal cells form a network in the inter-sinusoidal spaces of the bone cavity that extends from the endosteum to the endothelial cell basement membrane of the sinusoids (19). The interstitia of this network support the growth and differentiation of B cells in close contact with long cytoplasmatic processes of stromal cells (20, 21). During the first stages of the development from multipotent progenitor cells to pre-B cells, the interaction with stromal cells through CD117-stromal stem cell factor (SCF) and soluble factors is indispensable (22). In addition to cytokines like interleukin (IL)-3 and granulocyte-macrophage colony-stimulating factor (GM-CSF), which support the maturation of the developing B cell precursors (23), the exclusive secretion of IL-7 is an indispensable requirement for B cell development (24). Accordingly, mice that lack IL-7 (25, 26), the IL-7-receptor-alpha (IL-7Rα) chain (27) or the common gamma-c (γc) chain (28) all show a block in B cell development at the pro-B cell stage. This results in a strong reduction of the pre-B cell population and, consequently, of the mature B cell pool in the periphery.

The purpose of the current study was to gain more insights into the role of stromal cells on early B cell development from early pro-B cell to pre-B cell stage during infection with *T. cruzi* and how this parasite is capable to interfere with the hematopoietic system leading to immunosuppression. Our results suggest that during experimental Chagas disease a depletion of mature peripheral B cells commences already in the bone marrow concomitant with a considerable reduction in B cell development and increased apoptosis mediated by the changes in the stromal cell compartment.

## Materials and Methods

### Mice

C57BL/6J mice were bred in the animal facility of the Max-Planck-Institute for Immunobiology and Epigenetics (Freiburg, Germany). Acidified water (pH 3.0) and food were provided *ad libitum*. All mice were kept in individually ventilated filter cages. For production of parasites CB17 SCID mice were used (1). All experiments were conducted according to the German animal protection laws and were approved by the Animal Research Ethics Board of the regional council Freiburg (Germany) under the file number 35-9185.81/G-98/69.

### Parasites

Master stocks of the Tulahuen strain of *T. cruzi* were kept cryopreserved (3). This strain is classified into TcVI (29). For any given infection experiment, parasites were produced in CB17 SCID mice, isolated from the blood, counted and diluted to the desired concentrations as previously described (30). In each experiment, 3–5 mice per group were infected with 75 or 500 blood trypomastigotes (31).

### Infection Studies

For *in vivo* experiments, mice were intraperitoneally infected with the given amount of blood trypomastigotes. At the indicated time points the parasitemia was checked microscopically. Animals were sacrificed by cervical dislocation and the spleen, and the bone marrow were isolated and kept in ice cold ISCOVES medium for further analysis. As uninfected controls (0 dpi), naïve sex- and age-matched mice were used.

### Flow Cytometry

Single cell suspensions were prepared and washed in ISCOVES medium. After centrifugation, erythrocytes were lysed in Red Cell Removal Buffer (RCRB; 156 mM NH_4_Cl, 10 μM EDTA, 1 mM Na_2_CO_3_) and FCS was subsequently added (3). Cells were counted and 10^6^ cells per sample were used for staining. Cells were washed twice in PBS containing 3% FCS and 0.1% NaN_3_ and were subsequently stained with optimal concentrations of anti-IgM, anti-IgD, anti-B220, anti-CD25, anti-CD21, anti-CD43, anti-Thy 1.2, anti-NK1.1, or anti-CD138 (all from BD Bioscience). Fluorochrome-labeled streptavidin and the apoptosis marker merocyanine (Sigma Aldrich, Munich, Germany) were incubated separately. Samples were subsequently acquired on a FACSCalibur (BD Bioscience) and analyzed using the CellQuest software (BD Bioscience).

### Quantitation of Cytokine Transcripts by RNase Protection Assay

Because of cell to cell action of secreted cytokines such as IL-7 or IL-3 in B cell development measurement on protein level was not suitable, therefore we focused on the gene expression level. For RNA extraction, bone marrow was isolated from mice at days 6, 10, 14, 21, and 32 after infection. Day 6 after infection was used as an infection-internal reference. After homogenization in solution D [4 M guanidinium thiocyanate (Sigma Aldrich), 0.5% N-laurosylsarcosine (Sigma Aldrich), 1 M sodium citrate (Merck, Darmstadt, Germany), 0.1 M 2-ME (Serva, Heidelberg, Germany)] suspensions were acidified in 2 M sodium acetate, purified by extractions with phenol/chloroform, precipitated with isopropanol, and washed twice in 70% ethanol. Air-dried RNA was dissolved in RNase-free H_2_O and stored at −70°C until used. To determine the concentration and purity of extracted RNA, the absorption was determined at 260 and 280 nm, and possible degradation was examined after migration on a 1.2% formaldehyde/agarose gel. For hybridization with radiolabeled antisense RNA, extracted RNA was completely dried in a vacuum evaporator centrifuge and dissolved in hybridization buffer (RiboQuant; BD PharMingen, San Diego, CA).

Unlabeled sense RNA for IL-3, SCF, GM-CSF, IL-7, and L32 and all reagents were supplied by BD PharMingen (RiboQuant). For the synthesis of radiolabeled antisense RNA probes, the final reaction mixture (20 μl) contained 120 μCi of [α-32P]UTP (3000 Ci/mmol; Amersham, Arlington Heights, IL); UTP (61 pmol), GTP, ATP, and CTP (2.75 nmol each); 100 nmol DTT; transcription buffer (1 ×) RNasin (40 U); T7 RNA polymerase (20 U); and an equimolar pool of the template set. After 1 h at 37°C, the reaction was terminated by incubation with DNase (2 U) for 30 min at 37°C. Probes were purified by extractions with phenol/chloroform and precipitated with ethanol. Air-dried probes were dissolved (3 × 10^5^ cpm/μl) in hybridization buffer and added (2 μl) to tubes containing extracted RNA from bone marrow of *T. cruzi*-infected mice dissolved in 8 μl of hybridization buffer. Samples were overlaid with mineral oil, heated to 90°C, and incubated at 56°C for 16 h. Single-stranded RNA was then digested by addition (100 μl) of RNase A (80 ng/μl) and RNase T1 (250 U/μl) in RNase buffer. After incubation for 45 min at 30°C, samples were treated for 15 min at 37°C with 18 μl of a mixture of proteinase K (0.3 mg/ml), yeast RNA (0.06 mg/ml), and proteinase K buffer to stop digestion. dsRNA was isolated and precipitated as above, dissolved in loading buffer, and electrophoreses was performed in standard 6% acrylamide/8 M urea sequencing gel. Dried gels were placed on BioMax film (Kodak, Rochester, NY) with intensifying screens and were developed at −70°C for 72 h.

To quantify the relative amount of gene expression, gels were scanned and the respective bands were densitometrically evaluated with an Image Analysis System (NIH 1.52; National Institutes of Health, Bethesda, MD). Resulting densities of the genes of interest were standardized against L32.

### *In vitro* Studies

For the examination of stromal cells, we isolated the whole bone marrow of both femurs from uninfected and infected mice by flushing with a 1 ml syringe into ice cold ISCOVES medium. For any given day post infection bone marrow obtained from uninfected mice were used as control. Erythrocytes were eliminated with RCRB and the remaining bone marrow cells were washed and cultivated for 3 days (5% CO_2_, 37°C). Non-adherent cells were removed and the adherent cells were incubated for additional 3 days. Cultures of stromal cells were checked microscopically and images were taken by a computer aided camera.

Functional analysis of stromal cells was subsequently conducted. For the examination of B cell development on stromal cells, B220^+^IgM^−^ B cell precursors were sorted from bone marrow cell suspensions of uninfected mice with a MoFlo cellsorter. Sorted precursors were incubated with stromal cells isolated from uninfected and infected mice. After 3 days, non-adherent cells were collected, stained and analyzed by flow cytometry.

To examine the influence of different stimuli on cellular stress, stromal cells of naïve mice were prepared and incubated for 4 days with serum of naïve or 14 days infected C57BL/6 wild-type mice (diluted 1:6), L-arginine (Sigma Aldrich), the arginase inhibitor N-hydroxy-L-arginine (NhLA) (Sigma Aldrich), and IL-4 (Peprotech, Hamburg, Germany). Subsequently, a cellular stress assay was performed (WST-1; Roche) following the manufacturer's instructions (32).

### Statistical Analysis

Quantifiable data are expressed as the means of individual determinations and standard deviations. Statistical analysis was performed using, dependent on the data analyzed, Wilcoxon signed rank test, Mann–Whitney U test, or the two-tailed Student's *t*-test in Graphpad Prism (Graphpad Software, San Diego, CA.).

## Results

### B Cell Reduction in the Spleen

**Figure 1:**
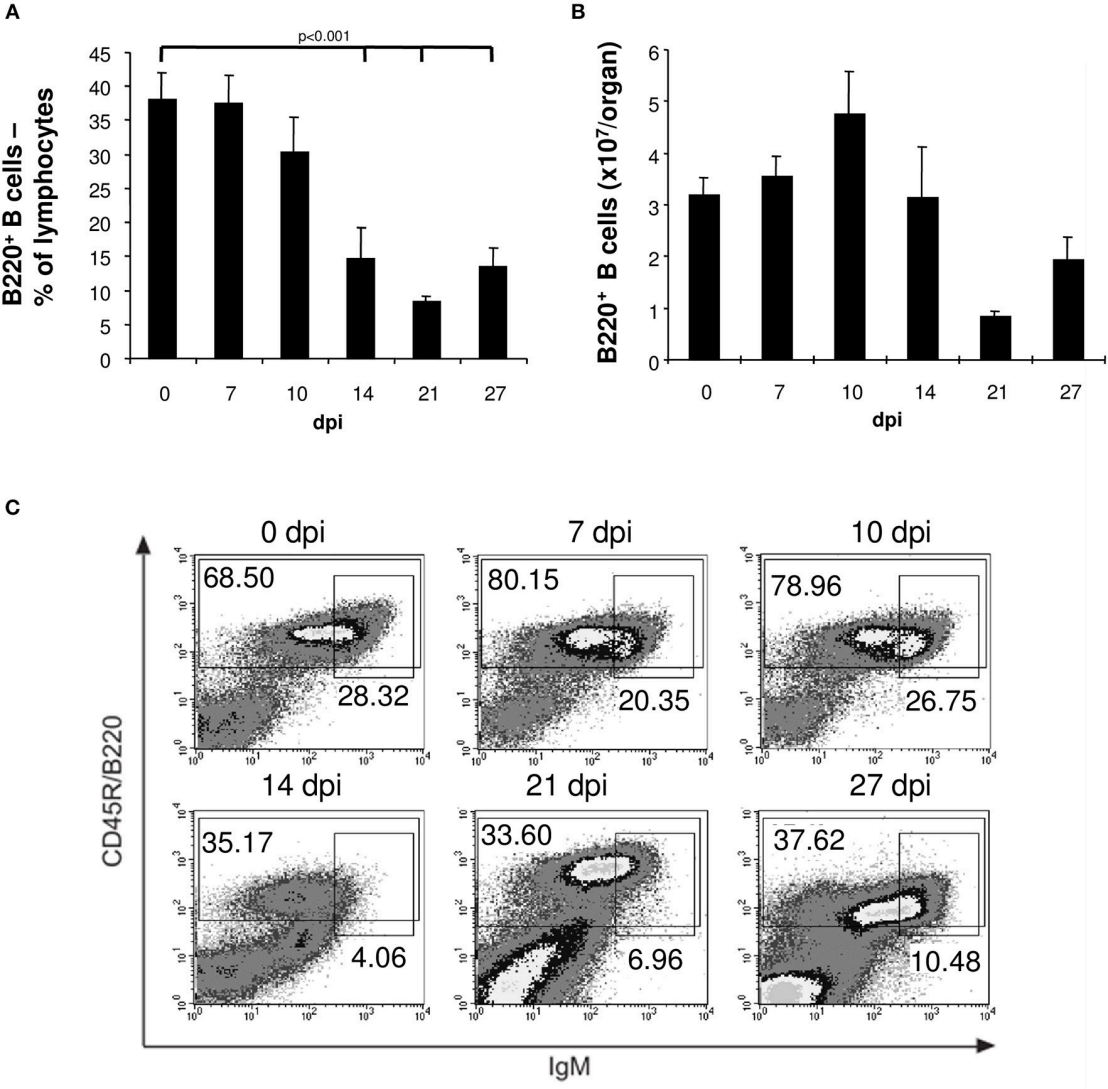
Reduced numbers of mature and transitional B cells in spleens of *T. cruzi*-infected mice. C57BL/6 mice were infected with 75 *T. cruzi* blood trypomastigotes i.p. (infections with 500 *T. cruzi* blood trypomastigotes show comparable results). At the indicated time points, spleen cells were analyzed by flow cytometry. **(A)** Frequency and **(B)** absolute numbers of B220^+^ B cells. **(C)** Representative flow cytometric analysis of mature B220^+^ IgM^dull^ and transitional immature B220^+^ IgM^hi^ B cells. Data represent mean ± SD of pooled results from three independent experiments each comprising 5 mice per time point. Statistical analysis was performed using two-tailed Student's *t*-test.

During the first 10 days of the acute phase of infection with *T. cruzi* trypomastigotes flow-cytometric analysis revealed an unchanged frequency of 40% B cells in the spleen of C57BL/6 mice (Figure 1A). During the further course of infection we observed splenomegaly (data not shown) accompanied by an increase in the total numbers of B lymphocytes (Figure 1B), T cells, NK cells, and macrophages (data not shown). However, after day 14 post infection the frequency of splenic B cells significantly dropped to 10% (Figure 1A) also resulting in a significant reduction of the total number of B cells (Figure 1B). In contrast to B cells, the frequencies and numbers of T cells, NK cells, and macrophages increased during the course of infection (data not shown). By flow-cytometry we show that the majority of the B cells were B220^+^ bright between day 0 and 10 post infection and became B220^+^ dull on day 14. The population of immature, transient B cells (B220^+^, IgM^+^ high) was reduced in the further course of infection reaching the lowest frequency 14 days post infection (Figure 1C). This reduction of B cells on day 14 pi was accompanied by an increased parasitemia in blood of infected C57BL/6 mice starting after day 14 pi with a maximum at day 21 pi, decreasing thereafter till day 35 pi to undetectable numbers (3).

**Figure 2:**
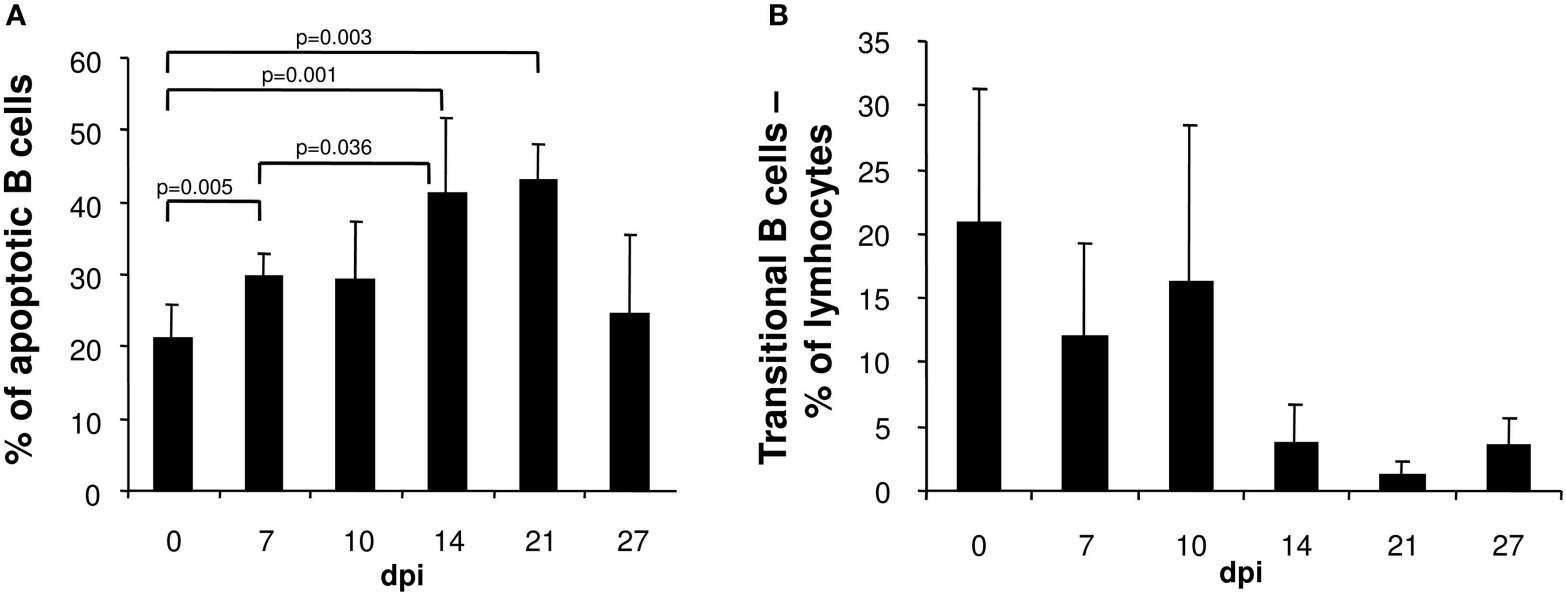
In the spleen of *T. cruzi*-infected mice the frequency of apoptotic B cells is elevated whereas the proportion of transient B cells is reduced. C57BL/6 mice were infected with 75 *T. cruzi* blood trypomastigotes i.p. At the indicated time points, spleen cells were analyzed by flow cytometry. **(A)** Frequency of merocyanine^+^ apoptotic B220^+^ cells. **(B)** Proportion of B220^+^IgM^hi^ transitional B cells. Data represent mean ± SD of pooled results from three independent experiments each comprising 5 mice per time point. Statistical analysis was performed using two-tailed Student's *t*-test.

The loss of immature transient B cells in the spleens of *T. cruzi*-infected mice could be attributed to apoptosis. Whereas, the frequency of apoptotic T cells, NK cells and macrophages in the spleen were unaltered during the course of infection (data not shown), the proportion of apoptotic B cells significantly increased after infection with a maximum at day 14 post infection (Figure 2A). Thereafter, the frequency of apoptotic B cells normalized. The lost B cell population was not replenished from the bone marrow, because the proportion of transitional B220^+^IgM^hi^ B cells migrating from the bone marrow to the spleen declined from day 14 post infection on (Figure 2B). Hence, in *T. cruzi*-infected mice, B cell depletion is not only due to apoptosis in the spleen, but also by the reduction of B cells in the bone marrow.

### B Cell Reduction and Loss of Stromal Cell Integrity in the Bone Marrow of *T. cruzi*-Infected Mice

**Figure 3:**
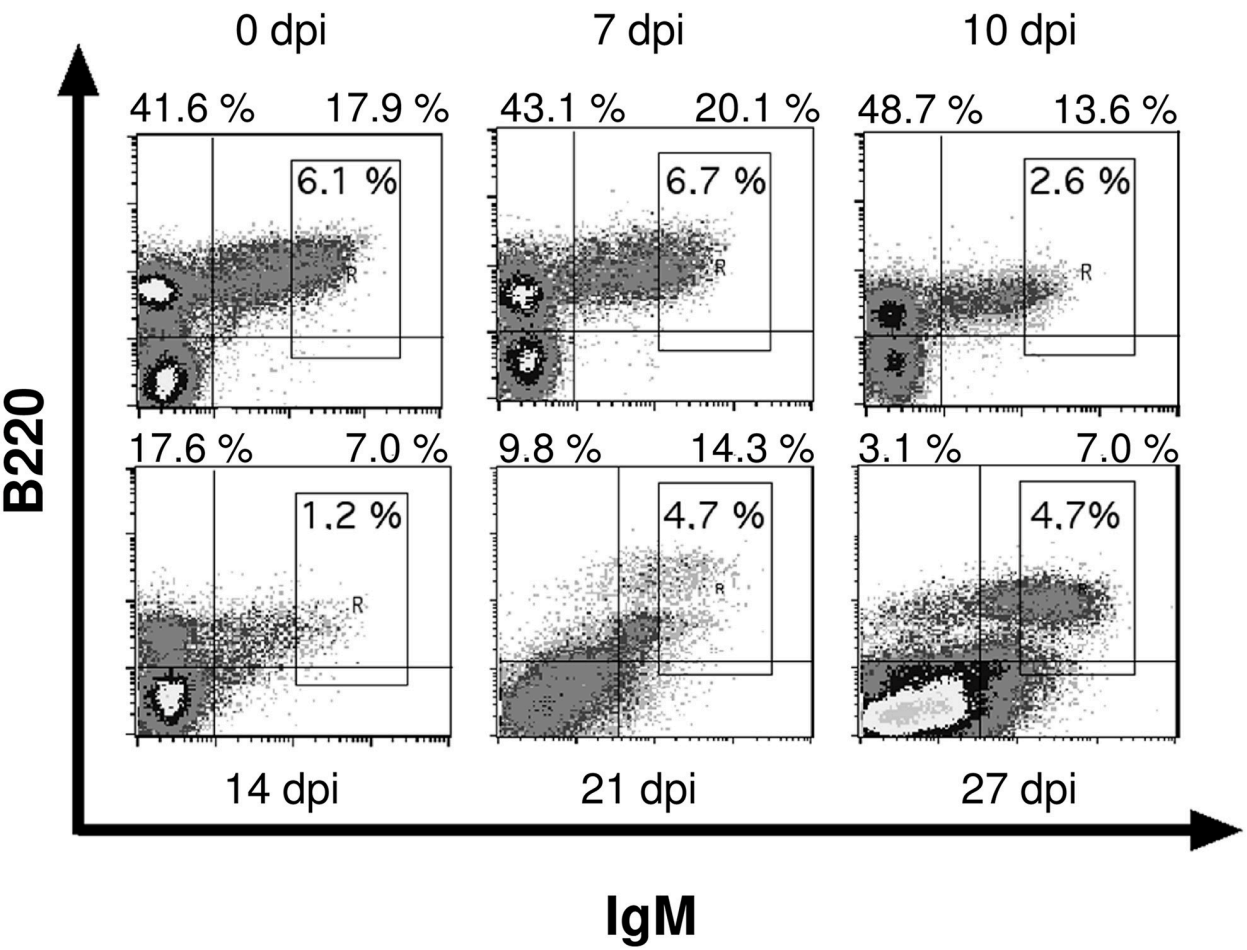
Infection with *T. cruzi* leads to reduced numbers of immature B cells in bone marrow. C57BL/6 mice were infected with 75 *T. cruzi* blood trypomastigotes i.p. (infections with 500 *T. cruzi* blood trypomastigotes show comparable results). At different time points, bone marrow immature B220^+^ IgM^+^ B cells were analyzed by flow cytometry. Representative plots from three independent experiments with 5 mice per experiment are shown.

**Figure 4:**
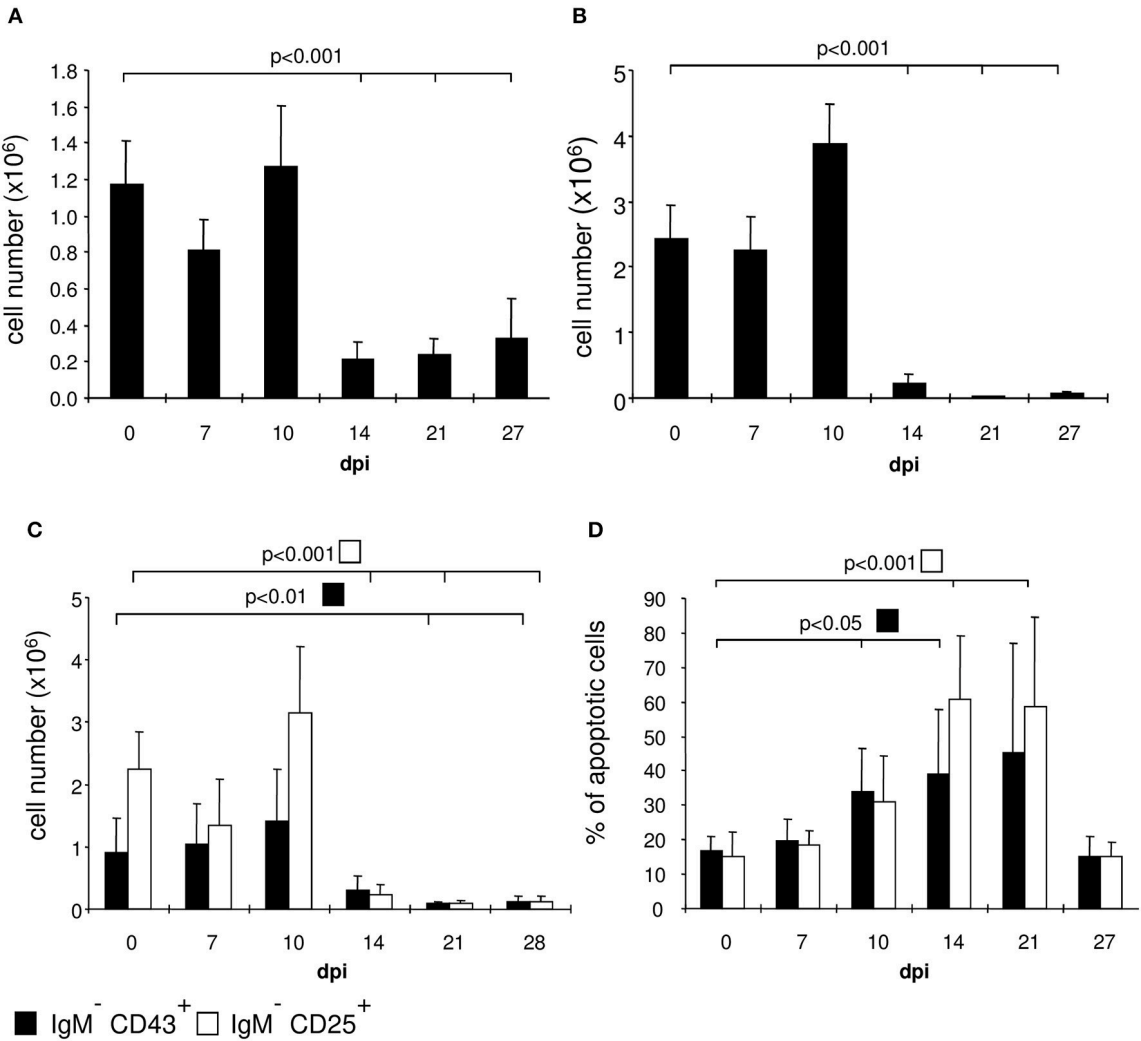
*T. cruzi* infection induces apoptosis of pro- and pre-B cells. C57BL/6 mice were infected with 75 *T. cruzi* blood trypomastigotes i.p. (infections with 500 *T. cruzi* blood trypomastigotes show comparable results). At the indicated time points we calculated the absolute numbers of **(A)** IgM^+^ B cells, **(B)** IgM^−^ precursors, and **(C)** the absolute numbers and **(D)** proportion of apoptotic CD43^+^ pro- and CD25^+^ pre-B cells. Data represent the mean ± SD of pooled results from three independent experiments each comprising 5 mice per time point. Statistical analysis was performed using two-tailed Student's *t*-test.

We examined the bone marrow of infected mice by flow cytometry and observed a severe hypoplasia with a strong reduction of IgM^+^ immature B cells after 10 days post infection (Figures 3, 4A). These cells undergo apoptosis (data not shown) accompanied by a reduction of IgM^−^ B cell precursors (Figures 3, 4B). Among these precursors, the absolute numbers of B220^+^ CD25^+^ pre- and B220^+^ CD43^+^ pro-B cells were significantly decreased from day 10 or 14 on, respectively (Figure 4C). Vice versa, pre- and pro-B cells underwent apoptosis significantly increasing after day 10 or 14, respectively (Figure 4D). Remarkably, apoptosis of both B cell precursor populations was abolished as of day 27 post infection.

**Figure 5:**
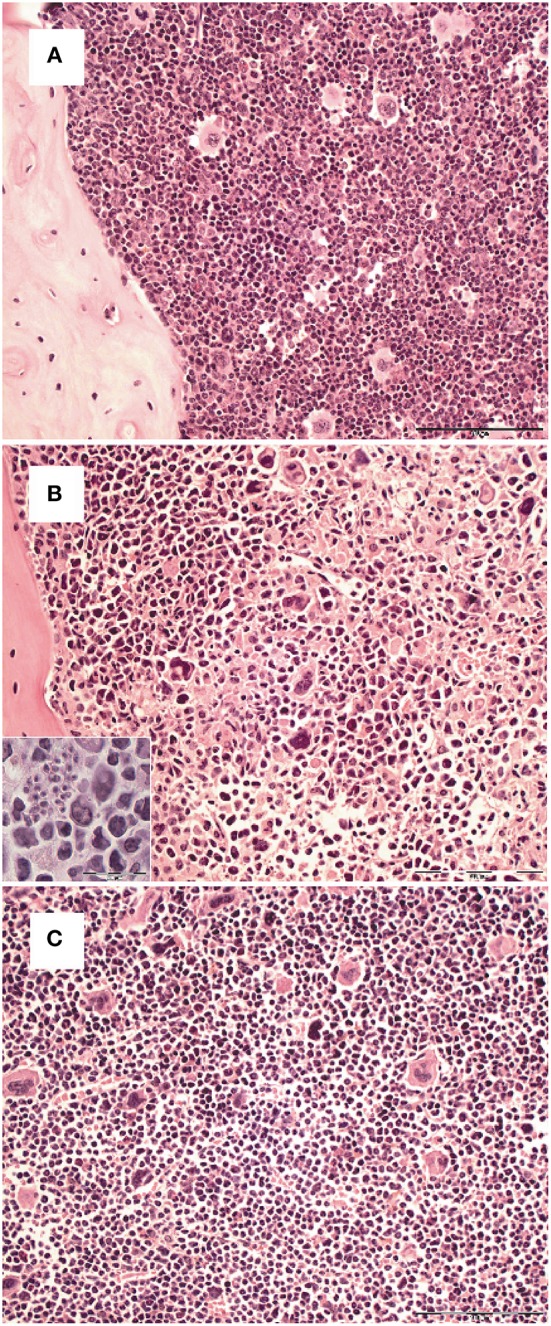
Transient loss of bone marrow stroma integrity during the course *T. cruzi* infection. C57BL/6 mice were infected with 75 *T. cruzi* blood trypomastigotes i.p. **(A)** Histopathological analysis of H&E stained sections of formalin-fixed and paraffin-embedded bones from hind legs of uninfected **(A)** and infected mice (**B**: 14 dpi; **C**: 35 dpi). Representative micrographs of one out of 5 mice are shown. (Bar, 100 μm. Inset, intracellular parasites in stromal cells; bar, 50 μm).

**Figure 6:**
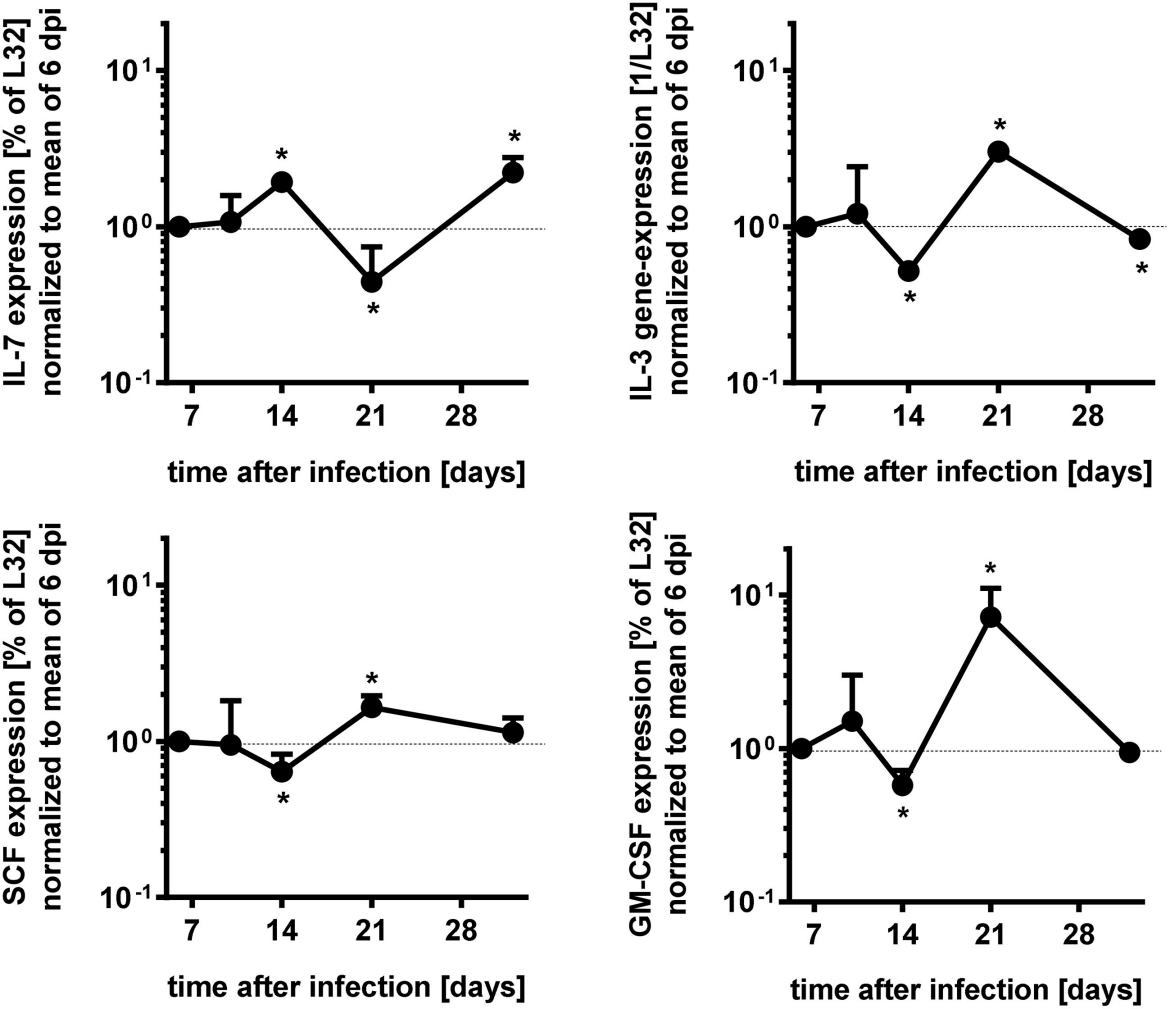
Modulation of gene expresssions during acute *T. cruzi* expression of bone marrow cells. Gene expression of *Il7, Il3, Gm-csf*, and *Scf i*n the bone marrow of infected mice were measured by RNase protection assay to time course dependent expressions. The values were first normalized to the expression of gene *L32* and second to the mean of the data of 6 dpi. Data represent mean ± SD of pooled results from two independent experiments each comprising 3 mice per time point, infected with 500 *T. cruzi* blood trypomastigotes i.p. Shown are the mean values and the upper error bars. Statistical analysis was performed using Wilcoxon rank sum test for significant differences. ^*^p > 0.05.

Survival and development of B cell precursors is dependent on the interaction with stromal cells of the bone marrow. They interact through the binding of hyaluronic acid to CD44 on pro-B cells, expression of stem cell factor (SCF) and c-kit as well as via cytokines released from stromal cells (23, 33). Particularly, IL-7 is an essential growth factor for late pro- and early pre-B cells (33), in addition also IL-3 and GM-CSF support B cell development (23, 34). Initial histopathological analyses of the bone marrow of *T. cruzi*-infected animals revealed a different density of stromal cells (Figure 5). Whereas the stroma of uninfected mice (Figure 5A) was dense, the stroma of mice infected appeared to be disintegrated at day 14. Of note, *T. cruzi*-infected cells were present in the bone marrow of these mice (Figure 5B inset), During the further course of infection, the histological appearance of the bone marrow recovered and the density of the stromal network infection was comparable to uninfected mice at day 35 (Figure 5C). Because IL-7 is an important stromal cell-derived factor for B cell development, besides SCF, IL-3, and GM-CSF we next analyzed gene expression of *Gm-csf*, *Scf*, *Il3*, and *Il7* in bone marrow of *T. cruzi*-infected mice. For analyzing the modulations of gene expression during infection, we have chosen day 6 pi as an infection-internal reference on that no alterations in pro- and pre-B cell numbers or apoptotic rates in comparison to the naïve state were detectable (see Figures 4C,D). Concomitant with the stromal breakdown after 14 days post infection, *Il3, Gm-csf*, and *Scf* expressions were significantly diminished on day 14 pi, followed by a decrease of *Il7* gene expression on day 21 pi, but the factors were restored later during infection (Figure 6). In summary, the restriction of B cell development during experimental Chagas disease was accompanied by a loss in stromal cell integrity and reduction of stromal cell-derived IL-3, GM-CSF, and IL-7 and a reduction of SCF expression on stromal surface.

### Impaired Capability of Stromal Cells From *T. cruzi*-Infected Mice to Promote B Cell Development *in vitro*

**Figure 7:**
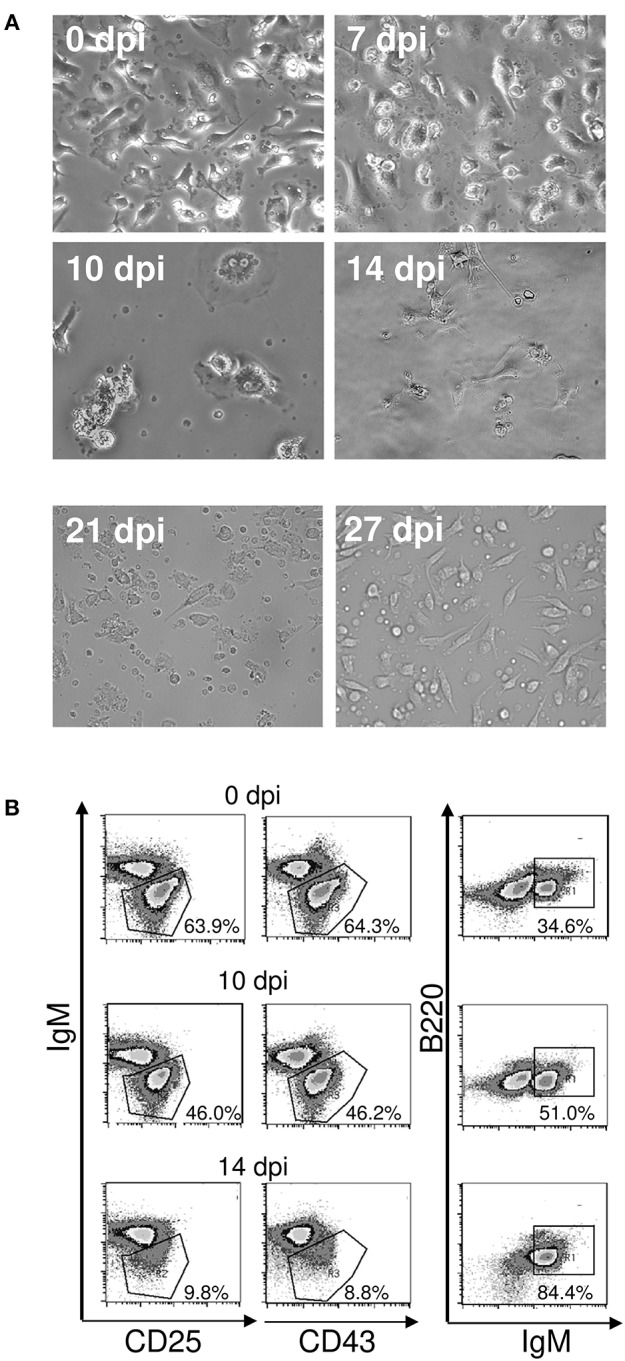
**(A)** Loss of function of bone marrow stromal cells during acute *T. cruzi* infection. C57BL/6 mice were infected with 75 *T. cruzi* blood trypomastigotes i.p. Bone marrow isolated from femurs of uninfected and infected mice were cultivated after removal of non-adherent cells. Representative photomicrographs of stromal cell cultures of 15 mice are shown. (0–10 d.p.i., magnification 400x; 14–27 d.p.i., magnification 300x). **(B)** Maintenance of pro- and pre-B cells. B220^+^IgM^−^ B cell progenitors were isolated by FACS and incubated with adherent bone marrow stromal cells from uninfected and infected mice. After 3 days, the frequencies of B220^+^ CD25^+^ pre-, B220^+^ CD43^+^ pro-, and B220^+^IgM^+^ immature B cells were analyzed by flow cytometry. Shown are representative plots from two independent experiments with 5 mice per experiment.

Subsequently, we evaluated the integrity of the stroma during infection with *T. cruzi* after *ex vivo* cultivation of bone marrow stromal cells for 72 h. In microscopy, the stromal cell population of the bone marrow was able to reach confluency until 7 days post infection but collapsed thereafter (Figure 7A). Importantly, the integrity of the stromal cell function was recovered on day 27 of infection. Hence, a stromal cell breakdown after infection with *T. cruzi* may account for the arrest in the development of B cells.

As a next step, we addressed the question whether the stromal cell breakdown during infection with *T. cruzi* was responsible for the diminshed B cell development. To this end, we co-cultivated sorted B220^+^IgM^−^ B cell precursors from uninfected mice on stromal cells of uninfected and infected mice. Incubation on stromal cells isolated from uninfected mice and from mice infected for 7 (data not shown) or 10 days similarly promoted B cell development as measured by the frequencies of pro- and pre B cells (Figure 7B). In contrast, very few B220^+^ CD25^+^ pre- and B220^+^ CD43^+^ pro-B cells could be recovered from co-cultures with stromal cells isolated from mice infected with *T. cruzi* for 14 days. Moreover, more than 80% of the analyzed cells represent immature B cells, a transitory state of transitional B cells (21)—indicating that stromal cells are inactive and only late pre-B cells could differentiate in the absence of a functional stroma (33). In the absence of stromal cell function, the replenishment of the B cell precursors is interrupted, resulting in diminished immature B cells. These results are in concordance with our *in vivo* findings (see Figure 1C), where especially the proportion of transitional B cells was largely decreased on day 14 pi.

**Figure 8:**
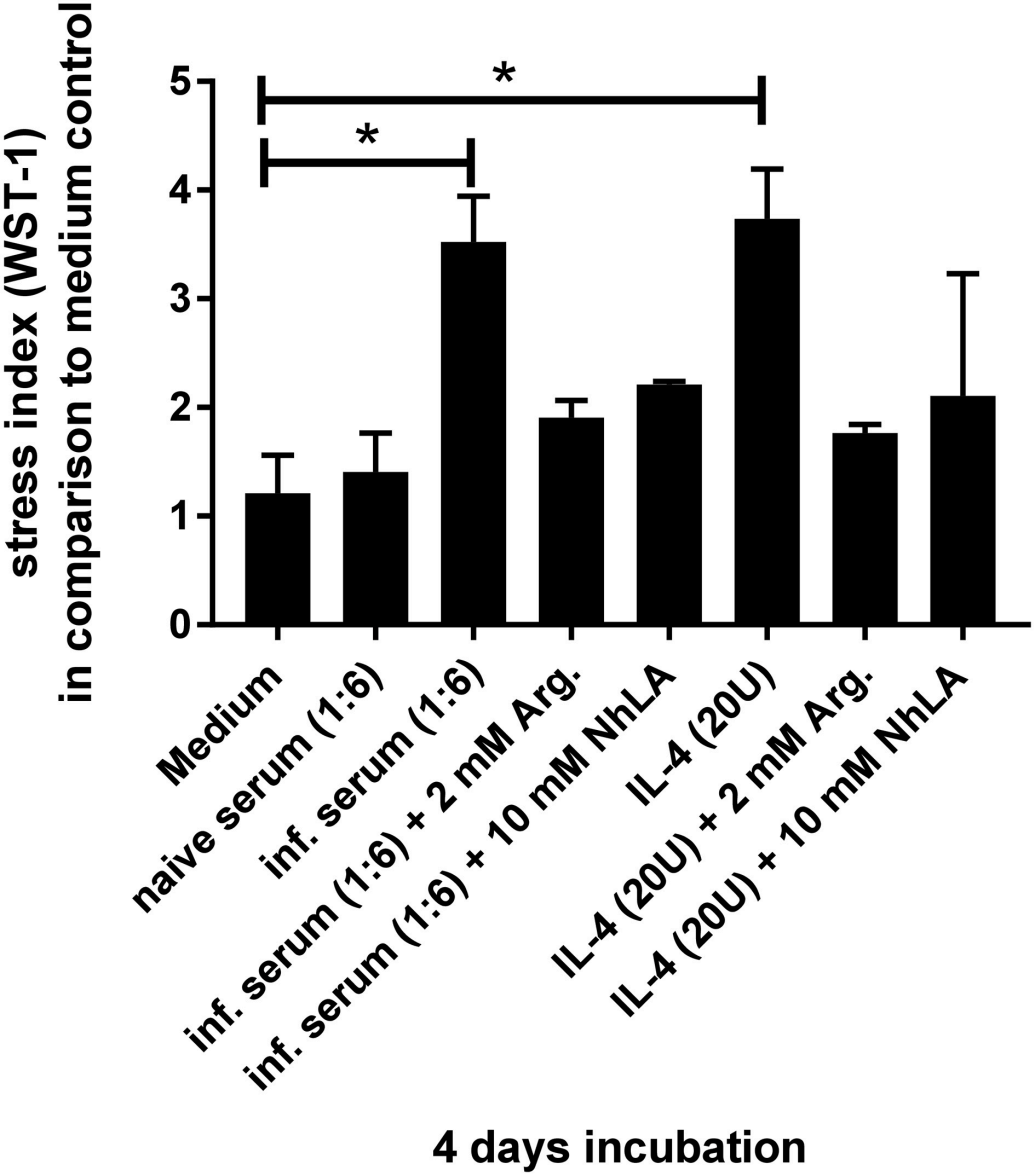
Examination of cellular stress induced by different stimuli. The designated stimuli were incubated for 4 days with stromal cells of naïve C57BL/6 wild-type mice and subsequent measurement of metabolized tetrazolium salt to formazan. The data were analyzed for significances in comparison to medium control with six independent samples, using the non-parametric Mann–Whitney *U* test. ^*^p > 0.05.

It has been reported that L-arginine has a major impact on the differentiation of B cells (35). In this instance, arginase-overexpressing mice have a comparable phenotype to *T. cruzi*-infected animals with respect to a block in B cell development (36). Because *T. cruzi* infection results in an elevated induction of the L-arginine-consuming enzymes nitric oxide synthase 2 (NOS2) and arginase-1 by the cytokines IFN-γ and IL-4, respectively (1, 37–40) we were wondering whether incubation with cytokine-containing serum of *T. cruzi*-infected mice has an effect on stromal cells isolated from uninfected mice. In contrast to medium alone and serum of uninfected animals, serum derived from infected mice induced a strong stress response in stromal cell cultures (Figure 8). Supplementation with L-arginine or treatment with the arginase inhibitor NhLA was able to rescue this phenotype. Furthermore, the addition of IL-4 as an inducer of arginase-1 stromal cells showed elevated metabolic rates. Again, supplementation with L-arginine or treatment with NhLA resulted in metabolic rates, comparable to cultures incubated with medium alone. Together, our results point to inflammation-induced consumption of L-arginine as a putative mechanism responsible for the transient considerable reduction in B cell development during acute experimental Chagas disease.

## Discussion

In the acute phase of an infection with *T. cruzi*, B cell activation results in the production of non-specific antibodies that fail to bind parasite antigens (41). Moreover, B and T cells display an impaired reactivity toward polyclonal activators (42, 43) and a depression of humoral responses to both, T cell-dependent (44) and -independent antigens (45), illustrating the immunosuppressive consequences of *T. cruzi* infection. Hence, in Chagas disease a continuous polyclonal activation of lymphocytes ends up in anergy of these cells (10). In addition, polyclonal activation induces apoptosis thus causing a reduction in B cell numbers (35, 46, 47). In the present study, we observed increased apoptosis of mature B cells in the spleen of *T. cruzi*-infected mice. Importantly, the amount of transitional B cells that come from the bone marrow was also reduced, thus explaining why the loss of B cells in the spleen was not replenished. Therefore, we examined the development of B cells in the bone marrow by flow cytometry, and also the numbers of pro-, pre-, and immature B cells were significantly diminished. Hence, the increased apoptosis of B lymphocytes in the spleen (48) appears not to be the only cause of the peripheral B cell depletion. In addition to findings from Adriana Gruppi et al. (49, 50), describing immature B cell apoptosis by myeloid cell derived factors, we describe here that in experimental Chagas disease a transient reduction in B cell development in the bone marrow results in reduced numbers of transitional B cells in the spleen and therefore additionally contributes to the loss of peripheral B cells during acute infection.

Besides polyclonal B cell activation, also bone marrow hypoplasia has been described in bacterial (51, 52), fungal (53), viral (54–56), and parasitic infections (57) including *T. cruzi* (11). In general, bone marrow hypoplasia is associated to anemia, thrombocytopenia, and leukopenia in infected mice. In agreement, myeloblasts in the bone marrow are strongly reduced in *T. cruzi*-infected mice (11) suggesting a possible damage to the bone marrow stroma during infection. Because stromal cells in the bone marrow are indispensable for the proliferation and development of myeloid and B cell precursors (20, 58–64) we asked the question of whether infection with *T. cruzi* affects this function of bone marrow stromal cells. Concomitant with the impaired development of B cell precursors and immature B cells, we show that the integrity of the bone marrow stroma was affected during acute *T. cruzi* infection. B cell precursors, such as pro- and pre-B cells, interact with stromal cells via receptor-ligand complexes and cytokines (21, 58, 65). Stromal cells produce the key cytokine IL-7 which is absolutely required for the proliferation of B cell precursors (25, 27, 66, 67). Additionally, other factors such as the SCF, that is required for cell-cell interaction via CD117 (c-kit) on the surface of pro B cells, IL-3, and GM-CSF, also contribute to an optimal B cell precursor differentiation (23). Hence, the transiently reduced *Il3, Gm-csf*, *Scf*, and *Il7 gene* expression in the bone marrow of *T. cruzi*-infected mice in our study further points at a dysfunction of stromal cells impacting on B cell development. B cell development depends also as aforementioned on the direct interaction of precursors with stromal cells via receptors and ligands (23, 62). To examine the function of bone marrow stromal cells during infection, we cultivated enriched B cell precursors of uninfected mice on bone marrow stromal cells isolated from uninfected and infected mice. The population of B cell precursors did not expand after incubation with stroma of mice infected for 14 days and most cells recovered from these cultures were immature B cells. Thus, the stroma in the bone marrow of *T. cruzi*-infected mice lost the capacity to promote B cell differentiation. Because pre-B cells can differentiate into immature B cells *in vitro* only in the absence of IL-7 and stromal cells (68) our results clearly demonstrate that *T. cruzi* induces a dysfunction of bone marrow stromal cells which is responsible for transient diminished B cell development during the acute infection. This alteration in the development of B cells contributed to the overall loss of this lymphocyte population in the periphery.

In addition to this direct infection-induced effect, the hyperinflammation during acute Chagas disease may also lead to reduced viability of stromal cells by apoptosis as is described for cells facing nutritional deprivation (69). A strong mediator of this programmed cell death and subsequent anemia is TNF (70–72). In Chagas disease patients a profound anemia (73) and in some cases an additional thrombocytopenia can occur (74, 75). Together, the parasite as well as the host immune response appear to induce the loss of stromal cell function, which is in turn responsible for the diminished B cell development during acute Chagas disease. In contrast to TNF-dependent modulations, our own data point to IL-4 as a soluble factor in *T. cruzi*-infected mice that may contribute to stromal cell alterations during the acute phase of Chagas disease (Figure 8). In this context, IL-4 has already been identified as an inhibitor of the development of B cell precursors (76). Because IL-4 induces the activation of the enzyme arginase-1 which is known to promote stromal cell depletion with a concomitant block in B cell development (36) the IL-4/arginase-1 axis may contribute to the loss of bone marrow stromal cells during the acute phase of *T. cruzi* infection. Elderly mice that are more susceptible to a *T. cruzi* infection express elevated levels of arginase in contrast to the more resistant younger individuals (77). In addition, a *T. cruzi* antigen, cruzipain (78) is able to induce arginase-1 in macrophages and treatment with the arginase inhibitor NhLA leads to a dramatic decrease of amastigote growth (79). In this context, the enzymatic axis of NOS2 and arginase, competing for L-arginine, is important for control or exacerbation of *T. cruzi* infection (39). In the here presented study, we show elevated metabolic rates for stromal cells incubated with serum of infected mice or exogenous IL-4. We therefore hypothesize, that under inflammatory conditions arginase activity is induced leading to a deprivation of L-arginine. Because L-arginine deprivation is concomitant with the upregulation of argininosuccinate synthetase and lyase activity (80) and the tetrazolium salt in the here applied WST-1 assay is metabolized by the succinate tetrazolium dehydrogenase to formazan, our results point not to enhanced viability but to signs of cellular stress induced by L-arginine deprivation.

Together, our data highlight the complex interplay of *T. cruzi* with the immune system that presumably foster a timely immune evasion of the parasite during the acute phase of infection by reducing the number of antibody-producing cells in the periphery through a developmental diminishment of their precursors already in the bone marrow. The transient changes in stromal cell function and subsequent loss of B cell precursors may appear to contribute to establish the parasite in the host. However, restoring the stromal cell integrity during the acute phase of *T. cruzi* infection would experimentally proof that the inflammation-induced alteration of stromal cell function in the bone marrow really affects B cell development and subsequently effector responses against the parasite. Together, rescuing the B cells and its precursors during the acute infection may limit the risk of a life-long infection by *T. cruzi* and the subsequent sequelae in the chronic phase of Chagas disease.

## Author Contributions

UM performed the experiments, analyzed the data, and wrote the manuscript. GS conceived the project. HM conceived the project. GK analyzed the data. RC conceived and designed the experiments and wrote the manuscript. CH conceived and designed the experiments and wrote the manuscript.

### Conflict of Interest Statement

The authors declare that the research was conducted in the absence of any commercial or financial relationships that could be construed as a potential conflict of interest.
